# Predicting the effects of rare genetic variants on oncogenic signaling pathways: A computational analysis of HRAS protein function

**DOI:** 10.3389/fchem.2023.1173624

**Published:** 2023-04-21

**Authors:** Sadaqat Ali, Usman Ali, Adeem Qamar, Imran Zafar, Muhammad Yaqoob, Qurat ul Ain, Summya Rashid, Rohit Sharma, Hiba-Allah Nafidi, Yousef A. Bin Jardan, Mohammed Bourhia

**Affiliations:** ^1^ Medical Department, DHQ Hospital Bhawalnagr, Punjab, Pakistan; ^2^ Basic Health Unit, Punjab, Pakistan; ^3^ Department of Pathology, Sahiwal Medical College Sahiwal, Punjab, Pakistan; ^4^ Department of Bioinformatics and Computational Biology, Virtual University of Pakistan, Punjab, Pakistan; ^5^ Department of Life Sciences, ARID University-Barani Institute of Sciences Burewala Campus, Punjab, Pakistan; ^6^ Department of Chemistry, Government College Women University, Faisalabad, Pakistan; ^7^ Department of Rasa Shastra and Bhaishajya Kalpana, Faculty of Ayurveda, Institute of Medical Sciences, Banaras Hindu University, Varanasi, Uttar Pradesh, India; ^8^ Department of Food Science, Faculty of Agricultural and Food Sciences, Laval University, Quebec City, QC, Canada; ^9^ Department of Pharmaceutics, College of Pharmacy, King Saud University, Riyadh, Saudi Arabia; ^10^ Laboratory of Chemistry and Biochemistry, Faculty of Medicine and Pharmacy, Ibn Zohr University, Agadir, Morocco

**Keywords:** cancer, genetic variants, mutations, oncogenic signaling pathways, SNPs, nsSNP, HRAS

## Abstract

The HRAS gene plays a crucial role in regulating essential cellular processes for life, and this gene's misregulation is linked to the development of various types of cancers. Nonsynonymous single nucleotide polymorphisms (nsSNPs) within the coding region of HRAS can cause detrimental mutations that disrupt wild-type protein function. In the current investigation, we have employed *in-silico* methodologies to anticipate the consequences of infrequent genetic variations on the functional properties of the HRAS protein. We have discovered a total of 50 nsSNPs, of which 23 were located in the exon region of the HRAS gene and denoting that they were expected to cause harm or be deleterious. Out of these 23, 10 nsSNPs ([G60V], [G60D], [R123P], [D38H], [I46T], [G115R], [R123G], [P11OL], [A59L], and [G13R]) were identified as having the most delterious effect based on results of SIFT analysis and PolyPhen2 scores ranging from 0.53 to 69. The DDG values −3.21 kcal/mol to 0.87 kcal/mol represent the free energy change associated with protein stability upon mutation. Interestingly, we identified that the three mutations (Y4C, T58I, and Y12E) were found to improve the structural stability of the protein. We performed molecular dynamics (MD) simulations to investigate the structural and dynamic effects of HRAS mutations. Our results showed that the stable model of HRAS had a significantly lower energy value of −18756 kj/mol compared to the initial model of −108915 kj/mol. The RMSD value for the wild-type complex was 4.40 Å, and the binding energies for the G60V, G60D, and D38H mutants were −107.09 kcal/mol, −109.42 kcal/mol, and −107.18 kcal/mol, respectively as compared to wild-type HRAS protein had −105.85 kcal/mol. The result of our investigation presents convincing corroboration for the potential functional significance of nsSNPs in augmenting HRAS expression and adding to the activation of malignant oncogenic signalling pathways.

## 1 Introduction

The HRAS gene is identified as the Harvey rat sarcoma viral oncogene homolog and is responsible for encoding a GTPase protein of small stature that belongs to the RAS family. A plethora of cellular processes, comprising proliferation, differentiation, and survival, are subject to regulation by this particular intracellular signalling pathway (Rajalingam et al., 2007). Genetic alterations in RAS genes, notably in HRAS, are among the most common mutations detected in human cancers (Kawazu et al., 2013). The aberrant functioning of the HRAS protein instigates the activation of downstream signalling pathways, namely, MAPK/ERK and PI3K/AKT, which are critical in promoting cell proliferation and survival. Notably, these pathways are often disrupted in cancer, thereby underscoring their significant contribution to the disease’s pathogenesis (De Luca et al., 2012; Asati et al., 2016). Several studies have confirmed the involvement of HRAS mutations in various cancers, including bladder, colon, head and neck, lung, and thyroid cancers (Ngan et al., 2022). For instance, activating HRAS mutations have been found in up to 10% of thyroid cancers, linked with aggressive disease and poor prognosis (Garcia-Rostan et al., 2003).

Similarly, in bladder cancer, HRAS mutations have been detected in 1%–2% of cases and are associated with high-grade tumours and advanced disease (Nagata et al., 2016). Identifying and characterizing HRAS mutations are vital for cancer diagnosis and treatment. HRAS mutations may serve as biomarkers for cancer diagnosis or prognosis or as targets for cancer therapies to inhibit RAS signalling (Kompier et al., 2010). Research on HRAS and its role in cancer remains an active area of investigation, with ongoing efforts to identify new mutations and decipher their functional consequences.

The HRAS gene and its protein product have significant roles in tumour genesis by regulating fundamental cellular processes such as growth, differentiation, and viability. In its wild-type cellular context, the expression and activity of HRAS are under the precise control of several signaling pathways, especially the RAS-MAPK axis, which is vital for cellular differentiation and growth. However, genetic mutations in the HRAS gene can disrupt the balance of the RAS-MAPK pathway, leading to unregulated cellular proliferation and neoplastic transformation (Rezatabar et al., 2019; Ullah et al., 2022). Such HRAS mutations have been consistently observed in several types of cancer, including squamous cell carcinoma of the head and neck, bladder cancer, and thyroid carcinoma (Jefferies and Foulkes, 2001; Gilardi et al., 2020). Moreover, the involvement of HRAS in cancer is not restricted to the RAS-MAPK pathway alone, as it extensively interacts with other pivotal signaling pathways and cellular processes, including but not limited to the PI3K-AKT pathway, Wnt signaling pathway and cytoskeleton. These intricate interactions facilitate the promotion of a multitude of oncogenic processes, such as cell survival, invasion, and metastasis. Therefore, the role of HRAS in cancer is multifaceted and cannot be limited to a single pathway or mechanism (Rezatabar et al., 2019; Shorning et al., 2020). The intricate protein-protein interaction network between HRAS and its downstream effectors in cancer cells comprises several signaling molecules, kinases, and transcription factors that are essential for malignant transformation and disease progression. A comprehensive understanding of this network can provide crucial insights into the mechanisms of HRAS-driven cancer and facilitate the development of innovative therapeutic interventions targeted at HRAS and its downstream effectors (Khan et al., 2020; Odeniyide et al., 2022).

Proteins exhibit nsSNPswhich can result in alterations in the amino acid sequence. It is well-established that such modifications have been linked to the initiation and advancement of cancer (Masoodi et al., 2013; Wang et al., 2019). These variations can arise in genes involved in cell growth regulation, such as oncogenes or tumour suppressor genes. They may impede normal cellular processes such as cell division and programmed cell death, associated with malignant transformation. One of the prototypical oncogenes affected by nsSNPs is the RAS gene family, encompassing HRAS, which encodes small GTPases involved in signalling pathways that control cellular proliferation, differentiation, and survival (Khan and Bisen, 2013; Makrides et al., 2017). Mutations in RAS genes, including nsSNPs, can activate these pathways and subvert normal cellular regulation, thereby instigating tumorigenesis. HRAS regulates several essential cellular processes, including cell differentiation, division, and programmed cell death. Mutations in HRAS have been observed in various cancers, including bladder cancer, pancreatic cancer, and lung cancer. NsSNPs within the coding region of HRAS may yield deleterious mutations that impair the normal function of the HRAS protein (Backwell and Marsh, 2022). Identifying and characterisingIdentifying and characterising nsSNPs in cancer-associated genes, including HRAS, is critical for comprehending the mechanisms underlying cancer pathogenesis and devising personalized cancer therapies (Pang, 2018). Computational tools can aid in predicting the impact of nsSNPs on protein function and can facilitate a deeper understanding of the contribution of these genetic variations to cancer onset and progression (Jubb et al., 2017; Ahmad et al., 2022).

The main objective of our research is identifying and characterising nsSNPs in the HRAS gene and their potential impact on the structure and function of the HRAS protein. Specifically, we explore *in silico* approaches to predict the effects of rare genetic variants on HRAS protein function, identify the most deleterious nsSNPs, and investigate the consequences of these mutations on the stability, flexibility, and compaction of the HRAS protein using molecular dynamics simulations. In this study, we also analyze the binding energies of the wild-type and mutant HRAS protein with docked complexes to understand the potential impact of these mutations on the activation of oncogenic signalling pathways. Our research provides compelling evidence for the potential functional role of nsSNPs in up-regulating HRAS expression and contributing to the development of various types of cancers.

## 2 Material and methods

### 2.1 Collecting and preparing SNP data

The ensuing discourse delineates the origins and manipulation of Single Nucleotide Polymorphism (SNP) data garnered from sundry databases like dbSNP (https://www.ncbi.nlm.nih.gov/snp/), ENSEMBLE (https://ensemblgenomes.org/), SNP500 cancer (https://pubmed.ncbi.nlm.nih.gov/), GeneCards (https://www.genecards.org/), and UniPort (https://www.uniprot.org/), which are periodically refreshed with new information. In particular, the ENSEMBLE repository was availed to obtain the nucleotide and protein sequences germane to the HRAS gene as per the methods of earlier researchers (Buljan et al., 2018; Zafar et al., 2022). This undertaking holds the promise of research prospects for exploring genetic variations and their potential implications (Rajaram et al., 2001).

### 2.2 Prediction of deleterious nsSNPs

The SIFT tool (https://sift.bii.a-star.edu.sg/) was employed to forecast the impact of non-synonymous SNPs on the mutant protein (Bromberg and Rost, 2007). This technique bifurcated the SNPs into two categories, intolerant and tolerant, based on homologous alignment (Seal et al., 2014). Precisely, amino acids with normalization probabilities falling beneath the designated threshold value were ascertained as intolerant, whilst those with a tolerance index measuring over >0.05 were considered tolerant (Dakal et al., 2017). The implications of this approach hold potential research prospects in assessing genetic variations and their resultant phenotypic outcomes (Pauls et al., 2013).

### 2.3 Structural homology-based approach: Coding of nsSNPs

The PolyPhen2 tool (http://genetics.bwh.harvard.edu/pph2/) was leveraged to predict the pernicious ramifications of nsSNPs on the structural and functional aspects of proteins. This prediction was based on the naive Bayesian algorithm, which involves classifying scores from 0 to 1. Mutations were partitioned into three categories, depending on the scores, with those possessing a score closest to 1 being identified as probably damaging and exhibiting a significant impact on protein structure. Implementing this tool could potentially open up research opportunities in the realm of genetic variability and its influence on protein conformation and function.

### 2.4 Categorization of functional nsSNPs

Identifying functional nsSNPs was executed with the aid of online servers, including SNP&GO (https://snps-and-go/), PhD-SNP (https://snps.biofold.org/), PROVEAN (https://bio.tools/provean), PANTHER (http://www.pantherdb.org/), and P-Mut (https://bio.tools/pmut). In particular, PhD-SNP relied on support vector systems to classify and depict the effects of non-synonymous SNPs on proteins. This method divided nsSNPs into deleterious or neutral categories (Li et al., 2006; Pauls et al., 2013). Furthermore, the ROVEAN tool was utilized to identify damaging SNPs by classifying mutations into deleterious or neutral depending on a threshold score of −2.5. At the same time, SNP&GO relied on the support vector machine algorithm (Sharma et al., 2022). Lastly, P-MUT employed the neural networking algorithm to segregate mutants into disease or neutral based on probability statistics of the sequences (Shinwari et al., 2022). These techniques could potentially unlock research opportunities in the sphere of genetic variations and their ramifications on protein function and structure (Singh et al., 2007).

### 2.5 Identification of nsSNPs on the coding area of protein

The SNP SnpEff and SnpSift (http://pcingola.github.io/SnpEff/) tool box was implemented to foretell the impact of nsSNPs on the coding region of the HRAS protein as per earlier researchers (Hossain et al., 2020). Apart from exhibiting the outcomes in conservation scores, the program also identified the protein homeostasis landscape (Han et al., 2020). The SNP effect used several software and tools to discover the propensity, including the aggregation tendency of the mutant via TANGO (https://switchlab.org/software/), amyloid propensity through WALTZ (https://switchlab.org/software/), and chaperone binding with LIMBO as per investigation of the earlier researcher (De Baets et al., 2012). These sophisticated methodologies can foster new research avenues for exploring the complex interplay between genetic variations and protein homeostasis.

### 2.6 Influence of non-synonymous mutations on protein stability (INPS)

The I-MUTANT 3.0 suit (https://bio.tools/i-mutant_suite) was utilized to prognosticate the impact of mutations on the stability of the HRAS protein, with the MUpro program (https://mupro.proteomics.ics.uci.edu/) being employed to validate the outcomes (Venkata Subbiah et al., 2020). Both servers rely on the same algorithm and are designed to evaluate the influence of mutations on protein stability, whether it is enhancing or diminishing it (Dehouck et al., 2011). Furthermore, the SRide server (http://sride.enzim.hu/) was leveraged to pinpoint the stabilizing residues of the native and mutant proteins (Kotha, 2010). The integration of these advanced computational techniques holds great promise for driving further research in protein stability and its response to genetic variations.

### 2.7 Conservational analysis of HRAS protein

The ConSurf server (https://consurf.tau.ac.il/consurf_index.php) was deployed to thoroughly analyze each amino acid’s conservation and evolutionary aspects in the HRAS protein (Kumar et al., 2021). The outcomes were presented as diverse conservation scores, ranging from 1 to 9. Scores from 1 to 3 corresponding to variable positions, while scores from 4 to 6 indicated amino acid positions that were moderately conserved. Furthermore, scores falling within the range of 7–9 represented highly conserved positions of amino acids. Utilizing this cutting-edge technology can pave the way for further protein evolution and conservation research, thereby expanding our knowledge of the complex interplay between genetics and protein function (Gourbal et al., 2018).

### 2.8 Molecular docking

We investigated the effects of mutations on the structure and function of the HRAS protein employing molecular docking analysis (Hossain et al., 2020). To achieve this, they acquired the three-dimensional configuration of the HRAS protein (PDB ID: 6MQT) from the Protein Data Bank. They transferred it into the MOE software (https://www.chemcomp.com/Products.htm) as per the method of the earlier researcher (Bhattacharya et al., 2017).

For the docking process, we excluded heteroatoms, ligands, and aqueous molecules from the structure. We conducted structural refinement employing definite parameters, such as energy minimization concerning 0.1 gradients, addition of hydrogen atoms, and utilization of the MMFF94X force field. We recognized an active site within the protein, encompassing a critical area of interacting residues.

Utilizing the MOE software, we performed molecular docking simulations for both standard and mutated molecules, storing the results in mdb format for further analysis (Ahmad et al., 2015). The highest-ranking postures underwent further refinement and calculation of binding free energies (ΔG) by employing the scoring function (GBVI/WSA dg). The scoring function is grounded on several molecular interactions, such as pi, hydrogen, and hydrophobic interactions. It presents a dependable scoring method that yields the docking score of the correct binding postures (Jin et al., 2023).

We meticulously surveyed the docked complex’s MOE database to understand the mode of binding interactions of the wild-type and mutated complex (Niranjan et al., 2021). This exploration enabled the research team to identify possible impacts of mutations on the protein’s structure and function, providing insightful discoveries for further investigations in the field.

### 2.9 Molecular dynamics (MD) simulation

We employed the Schrodinger 2021.2 software suite for their computational investigations (Kutzner et al., 2022)., The initial structures were drawn using Maestro 12.8 and subsequently ionized with Epik 3.2 program at a pH of 7.4 using Ligprep 3.4 (https://www.schrodinger.com/products/ligprep) as per the analysis of (Santana-Romo et al., 2020). This protocol was carried out to produce the requisite starting structures for molecular dynamics (MD) simulations.

We perform calculations to produce MD simulation trajectories across various intervals during the simulation run (Galindo-Murillo et al., 2015). After docking, the conformational study of three complexes was executed with the MacroModel 10.8 module (Kellici et al., 2019). This module employs a torsional sampling approach for all conformational searche:a Monte Carlo multiple minimum method (Li and Scheraga, 1987). The highest energy conformers were removed using a 21 kJ mol1 energy limit. Each conformer was reduced for a maximum of 2,000 steps using the Polak-Ribiere conjugate gradient technique, with a gradient convergence threshold of 0.001 kJ mol1 A˚ 1, and the OPLS3 force field was utilized for this process. The OPLS3 force field is advantageous for small molecules because it delivers accurate energy minimization potential functions (Harder et al., 2016).

The team employed MD simulation to appraise the stability of the enzyme-inhibitor complex and explore the conformational aspects of protein-ligand interactions (Amaral et al., 2017). The conformational variations and stability index of secondary structural components of the simulated complexes were assessed utilizing data reduction techniques such as root-mean-square deviation (RMSD), root-mean-square fluctuation (RMSF), the radius of gyration (Rg), and beta-factor values as per earlier researcher (Kumar et al., 2019). We employed a suite of computational software to perform molecular dynamics simulations and scrutinize the conformational aspects of protein-ligand interactions. They used diverse techniques to appraise the stability and conformational variations of the simulated complexes.

## 3 Results and discussion

### 3.1 Compilation of a single nucleotide polymorphism (SNP) library

We aimed to construct a comprehensive SNP database for the HRAS gene, a human gene that encodes for the protein HRAS (Hossain et al., 2020). The team utilized various bioinformatics tools and databases to achieve this goal, including the Ensemble genome browser, Gene card, Uniprot, and NCBI db-SNP (Phillips, 2009). These resources provide access to various genetic and protein-related information, which the team could use to identify and analyze SNPs in the HRAS gene (Kohl et al., 2015). Our analysis was on non-synonymous SNPs (nsSNPs), which are genetic variations that change the amino acid sequence of the protein encoded by the HRAS gene (Chai et al., 2022). These variations are more likely to impact the structure and function of the HRAS protein, potentially leading to changes in protein activity and, in turn, contributing to disease development.

**FIGURE 1:**
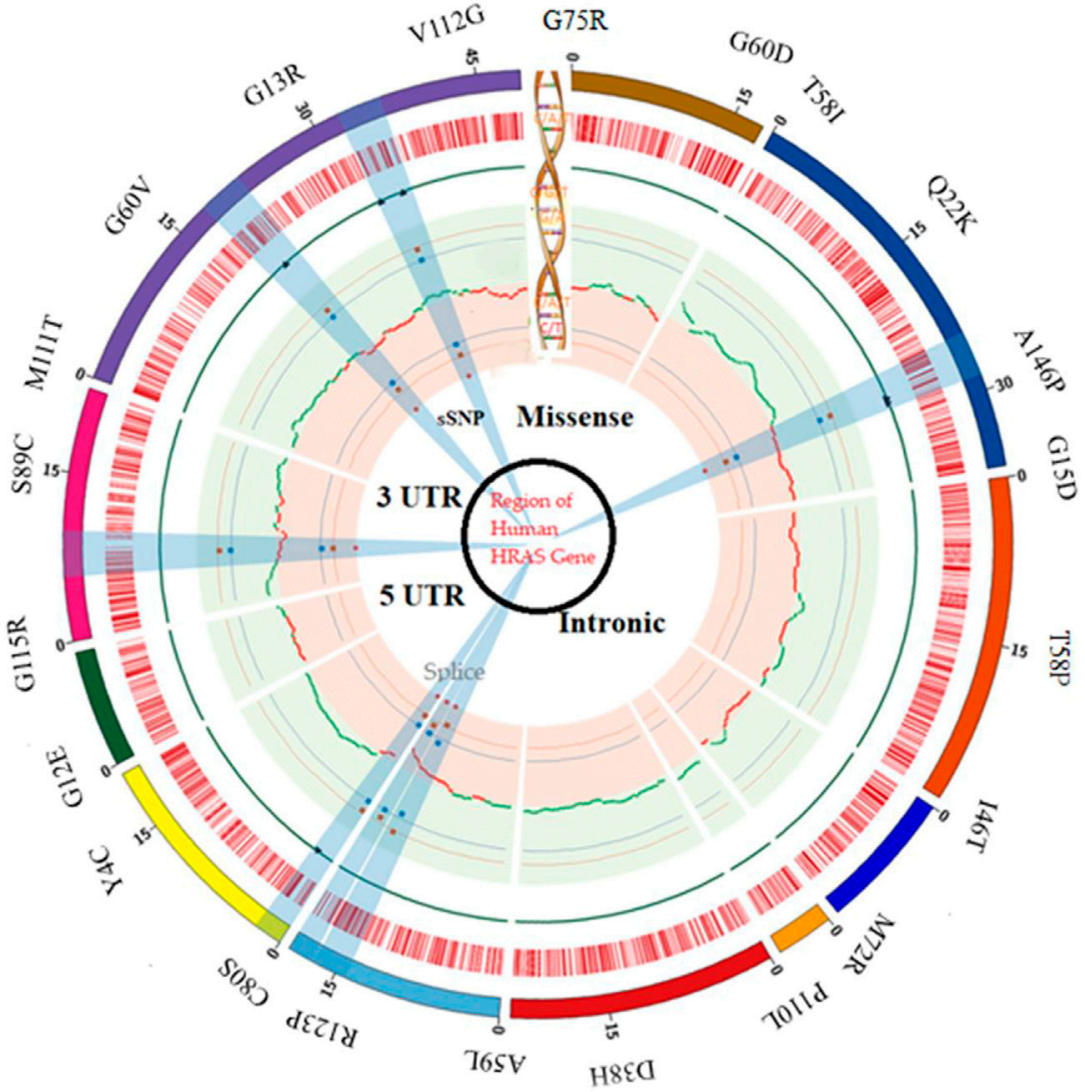
SNP distribution in a different region of the human HRAS gene.

We specifically looked for nsSNPs located within the HRAS gene’s exon region, which is the coding region translated into protein (Tarek et al., 2021). They identified the location and nature of each nsSNP and determined its frequency in different populations. However, during their analysis, we observed that many SNPs were located in the intron region of the HRAS gene (Estep et al., 2006). Intron regions are non-coding regions of the gene and traditionally were thought to be non-functional. However, recent studies have suggested that intronic SNPs could also have a role in regulating gene expression and splicing and may contribute to disease susceptibility (Meyer et al., 2008). Therefore, we also analyzed the potential impact of these intronic SNPs on HRAS gene expression and splicing as per earlier investigations (Vornholt et al., 2021). The distribution of SNPs in a different region of human HRAS gene is represented in Figure 1.

Our work involved using various bioinformatics tools and databases to map a comprehensive SNP dataset for the HRAS gene, focusing on non-synonymous SNPs in the exon region (Rivera et al., 2005). This dataset could provide valuable insights into the genetic variations contributing to disease development and help develop personalized medicine approaches (Hamburg and Collins, 2010).

### 3.2 Evaluation of non-synonymous (nsSNP) SNPs

We use the SIFT (Sorting Intolerant from Tolerant) algorithm to predict the impact of non-synonymous SNPs (nsSNPs) on the structure of the HRAS protein (Chai et al., 2022). The SIFT algorithm uses homologous protein sequences to determine which amino acid substitutions are likely to be tolerated and which are likely deleterious (Ng and Henikoff, 2001). The algorithm’s output is a tolerance index (TI) score, ranging from 0 to 1, with lower scores indicating a greater likelihood of harmful consequences. We submitted 50 nsSNPs to the SIFT algorithm for analysis (Savas et al., 2004). The of 23 SNPs were identified with TI scores ranging from 0 to 0.04, with 28 having a TI score of 0, indicating highly deleterious consequences. This means that these nsSNPs are likely to impact the structure and function of the HRAS protein significantly and the result matched with earlier research (Chai et al., 2022). Multiple SNPs had a TI score of 0.01, one had a score of 0.02, and one had a score of 0.004, suggesting only minor importance.

We noted a high frequency of substitutions involving cytosine and thymine or guanine and adenine, while substitutions involving adenine and thymine or adenine and cytosine were rare (Kim et al., 2019). This information could provide important insights into the mechanisms by which these nsSNPs affect HRAS protein function and may be helpful in developing targeted therapies for diseases caused by HRAS mutations as per the investigation of the earlier researcher (Chai et al., 2022). Overall, the use of the SIFT algorithm provides valuable information for understanding the impact of nsSNPs on the HRAS protein and could potentially contribute to the development of new treatments for diseases caused by HRAS mutations (Hossain et al., 2020).

### 3.3 Identification of functional modifications in coding nsSNPs

We describe the results of further analysis of 50 selected nsSNPs submitted to a server and utilized the PolyPhen2 algorithm to predict the impact of nsSNPs on protein structure. The algorithm provides a score between 0 and 1, with a higher score indicating a greater likelihood of deleterious consequences. Out of the 50 nsSNPs submitted, only 10 had a probabilistic score greater than 0.97, indicating that they were probably damaging nsSNPs. The total of 10 nsSNPs had scores higher than 0.83, which were classified as possibly damaging. We exactly identified seven nsSNPs (G75R, P34S, G60D, G60V, T58I, G60D, and A146P) with a maximum score of 1, indicating a high probability of being damaging. Most of the remaining mutations had scores in the range of 0.98 to −0.99. We also compared the results obtained from SIFT and PolyPhen2 and observed that ten nsSNPs were identified as common between the SIFT and PolyPhen2 analyses, despite using different methods to obtain results. The SIFT algorithm utilizes structural detail to yield results, while PolyPhen2 is based on structure and has shown a good correlation with the SIFT approach. Finally, we observed that most nsSNPs had a SIFT tolerance index of 0.00, indicating that they were highly intolerant to variation, while their PolyPhen2 scores were >0.90 and based on these findings, we concluded that these alterations may be responsible for disease.

### 3.4 Phenotypic impact of mutations

The SNP-effect tool assesses the potential effects of genetic variants on protein structure and function. We also evaluate the phenotypic impact of alterations within the HRAS molecule. Specifically, the approach evaluated chaperone binding propensity, aggregation propensity, and amyloid tendency. Chaperones are a class of proteins that assist in properly folding and assembly of other proteins. A protein’s chaperone binding propensity can provide insight into its stability and folding efficiency. Aggregation propensity refers to the tendency of proteins to form aggregates or clumps, which can interfere with proper cellular function. The amyloid tendency measures a protein’s ability to form amyloid fibrils, which are associated with several diseases, including Alzheimer’s and Parkinson’s and Cancer. The results of the SNP-effect analysis showed that the selected mutations had little to no impact on chaperone binding propensity, aggregation propensity, or amyloid tendency. However, it is essential to note that this approach only evaluates a limited set of characteristics and does not comprehensively assess all possible effects. The findings suggest that the selected mutations may have other potential impact on the structure and properties of the HRAS protein, which could have significant implications for biological organisms. Further experimental studies would be necessary to fully understand the impact of these mutations and their potential role in disease. Screening of most deleterious SNPs using different software’s are mentioned in Table 1; the HRAS gene encodes HRAS protein and plays an essential role in various cellular processes, including cell proliferation, differentiation, and survival. SNPs in the HRAS gene have been associated with multiple diseases, including cancer, and can impact protein structure and function. Therefore, identifying deleterious SNPs in the HRAS gene is crucial for understanding disease mechanisms and developing potential treatments.

**TABLE 1:** Screening of most deleterious SNPs by using different software.

No.	Variant ID	Alleles	Amino acid changes	SIFT	Polyphen2	Provean deleterious	P-mut disease
1	rs756190012	C/T	G75R	0.03	1	−7.893	0.69
2	rs730880460	C/A/T	G60V	0	1	−6.760	0.66
3	rs121917758	G/A	T58I	0	1	D −5.798	0.66
4	rs104894231	C/G/T	A146P	0	1	−4.427	0.53
5	rs730880460	C/A/T	G60D	0	1	−8.689	0.69
6	rs770492627	T/G	T58P	0	0.999	−5.797	0.53
7	rs755488418	A/C	M72R	0	0.999	−5.873	0.66
8	rs755322824	G/C	S89C	0	0.999	−4.711	0.64
9	rs764622691	T/C	Y4C	0	0.999	−6.949	0.66
10	rs730880464	C/G	R123P	0	0.998	−5.560	0.69
11	rs750680771	C/G/T	D38H	0	0.998	−6.461	0.66
12	rs1564789700	A/G	I46T	0	0.998	−4.596	0.64
13	rs1554885139	C/T	G15D	0	0.996	−5.631	0.63
14	rs1564789063	A/G	M111T	0	0.995	−4.376	0.64
15	rs121917757	G/A/T	Q22K	0	0.993	−3.341	0.62
16	rs917210997	C/T	G115R	0	0.993	−7.193	0.69
17	rs1370566417	A/T	C80S	0	0.992	−7.008	0.64
18	rs1204223913	G/A	G60V	0	0.99	−9.167	0.69
19	rs727504747	GC/AG	A59L	0	0.988	−4.756	0.69
20	rs1427823770	A/C	V112G	0	0.97	−6.172	0.67
21	rs104894228	C/A/G	G13R	0	0.975	−6.676	0.66
22	rs898057728	G/A/S	S65R	0	0.947	−4.536	0.67
23	rs727503094	GC/AG	G12E	0.01	0.942	−6.114	0.66

Screening potentially deleterious SNPs in the HRAS gene is essential to understanding the possible impact of genetic variations on the protein’s function (Chai et al., 2022). Several software tools have been developed to analyze the potential effect of SNPs on protein structure and function (Paniri et al., 2021). We applied SIFT, PolyPhen2, and SNP-effect to predict the impact of nsSNPs on the HRAS protein. The SIFT algorithm is a sequence-based tool that utilizes sequence homology to predict the potential effect of nsSNPs on protein function. SIFT analysis identified 23nsSNPs with TI scores ranging from 0.53 to 69, suggesting highly deleterious consequences. PolyPhen2 is a tool that combines sequence-based and structure-based predictions to predict the effect of nsSNPs on protein structure and function. We found that G75R, P34S, G60D, A59L, G60V, I46T, D38H, T58I, and A146P had a maximum score of 1, while most mutations had scores in the range of 0.98 to −0.99. A total of 10 nsSNPs were identified as standard between the SIFT and PolyPhen2 analyses, despite using different methods to obtain results.

The SNP-effect tool is a computational pipeline that assesses the effect of SNPs on protein properties, such as chaperone binding propensity, aggregation propensity, and amyloid tendency (Ji et al., 2021). We found that the selected alternate variants did not significantly impact these characteristics. However, they suggested that variations in protein structure and properties resulting from these SNPs could still significantly impact biological organisms. In summary, the use of multiple software tools to predict the effects of nsSNPs on the HRAS protein provides a more comprehensive understanding of the potential consequences of genetic variations, where earlier researchers (Hossain et al., 2020; Chai et al., 2022) also explore and indicate exact predictions. The SIFT and PolyPhen2 analyses identified several nsSNPs with a high likelihood of deleterious consequences. In contrast, the SNP-effect study showed that the selected nsSNPs did not significantly affect the protein’s chaperone binding, aggregation propensity, or amyloid tendency. These findings could have implications for understanding the role of HRAS mutations in developing various diseases.

### 3.5 Effect of mutation on stability of HRAS

The stability of a protein is crucial for its proper function, and destabilizing mutations can lead to the misfolding and aggregation of the protein, resulting in various diseases (Gámez et al., 2018). We explored the effect of different mutations on the stability of the HRAS protein and assessed using I-MUTANT and MUpro for final results and validations. The Delta Gibbs free energy (DDG) values were calculated to determine the stability of the protein, and a DDG value lower than 0 indicated a destabilizing mutation. The I-MUTANT (https://folding.biofold.org/i-mutant/i-mutant2.0.html) server predicted that most modifications can decrease the stability of the HRAS protein, with DDG values ranging from −3.21 kcal/mol to 0.87 kcal/mol. The most significant effect was observed with the I46T mutation, which had a DDG value of −3.21 kcal/mol. Only three mutations, Y4C, T58I, and Y12E, showed perfection in the structural stability of the protein. The MUpro server (https://mupro.proteomics.ics.uci.edu/) provided similar results, except for the Y4C, G12E, and T58I mutations, which showed a decrease in stability. The G15D mutation showed an increase in strength in the MUpro prediction, while I-MUTANT predicted a reduction in stability and the results are summarized in Table 2.

**TABLE 2:** Non-synonymous single nucleotide polymorphisms (nsSNPs) and their predicted effects on protein function.

Variant ID	Position	Wild type	Mutant	DDG value
rs730880460	60	G	D	−2.19
rs121917758	58	T	I	0.28
rs730880460	60	G	V	−1.22
rs764622691	4	Y	C	0.62
rs730880464	123	R	P	−1.13
rs750680771	38	D	H	−1.57
rs1564789700	46	I	T	−3.21
rs1554885139	15	G	D	−0.4
rs917210997	115	G	R	−0.98
rs369106578	123	R	G	−1.01
rs1204223913	110	P	L	−0.87
rs727504747	59	A	L	−0.32
rs104894228	13	G	R	−1.27
rs1564789552	64	Y	H	−0.94
rs727503094	12	G	E	0.87

The differences in predictions between the two servers can be attributed to their different calculation methods. I-MUTANT uses a support vector regression algorithm based on various structural properties of the protein, such as solvent accessibility and secondary structure, to predict the DDG values. In contrast, MUpro employs a neural network-based approach that incorporates sequence and structural information, as well as evolutionary conservation, to predict the effects of mutations on protein stability. The results of this study indicate that several mutations within the HRAS gene can destabilise the protein, potentially resulting in disease. Identifying these destabilizing mutations can provide insights into the molecular mechanisms of HRAS-associated diseases and may aid in developing new therapeutic strategies.

Our results indicated that most of the mutations decreased the stability of the HRAS protein, while only a few improved it. The mutation with the most significant effect on stability was I46T, with a DDG value of −3.21 kcal/mol. The I-MUTANT server predicted that 15 mutations, including Y4C, G12E, G13R, G15D, D38H, I46T, T58I, A59L, G75R, P34S, G60D, G60V, G115R, R123G, and R123P, decreased the stability of the protein, while Y4C, T58I, and Y12E improved it. The MUpro server provided similar results, except for Y4C, G12E, and T58I mutations, which showed decreasedd stability, contrary to the I-MUTANT prediction. Additionally, MUpro predicted I-MUTANT predicted a decrease inincreased stability for the G15D mutation, and I-MUTANT predicted decreased stability. The DDG values of most mutations ranged from −3.21 kcal/mol to 0.87 kcal/mol, indicating reduced protein stability with a DDG value lowers than 0. The results suggest that mutations can significantly affect the HRAS protein’s stability, which may impact the biological organism. Further research may be required to understand the specific effects of each mutation on HRAS protein stability and its overall impact on biological systems.

### 3.6 Conservation analysis

The ConSurf server (https://consurf.tau.ac.il/consurf_index.php) is a valuable tool for determining the evolutionary conservation of protein residues across a set of homologous sequences. The conservation scores of HRAS protein residues were analyzed using ConSurf to evaluate the impact of the 10 deleterious mutations on the protein structure and function. Out of the 10 deleterious mutations, 6 missense mutations (G13R, D38H, A59L, G60V, G60D, G115R, R123P, and R123G) were located in highly conserved regions (7–8–9). This finding suggests that mutations in these regions could significantly affect the function and structure of the HRAS protein. Additionally, G13R, D38H, and I46T were predicted to be exposed, while mutants such as A59L, G60V, G60D, Q60D, R123P, and R123G were expected to be functional and revealed mutations.

The conservation analysis showed that one mutation was located in a variable region (1–2–3) and one in an average part, indicating that these mutations may have a milder effect on the protein structure and function. These findings suggest that highly conserved regions of HRAS protein are more sensitive to mutations that could impact the protein function. These results provide insights into the functional and structural effects of the 10 selected deleterious mutations on the HRAS protein. By identifying the regions that are highly conserved and sensitive to mutations, this study can help researchers better understand the consequences of HRAS mutations and may lead to new treatments for diseases associated with HRAS mutations.

### 3.7 3D structures

**FIGURE 2:**
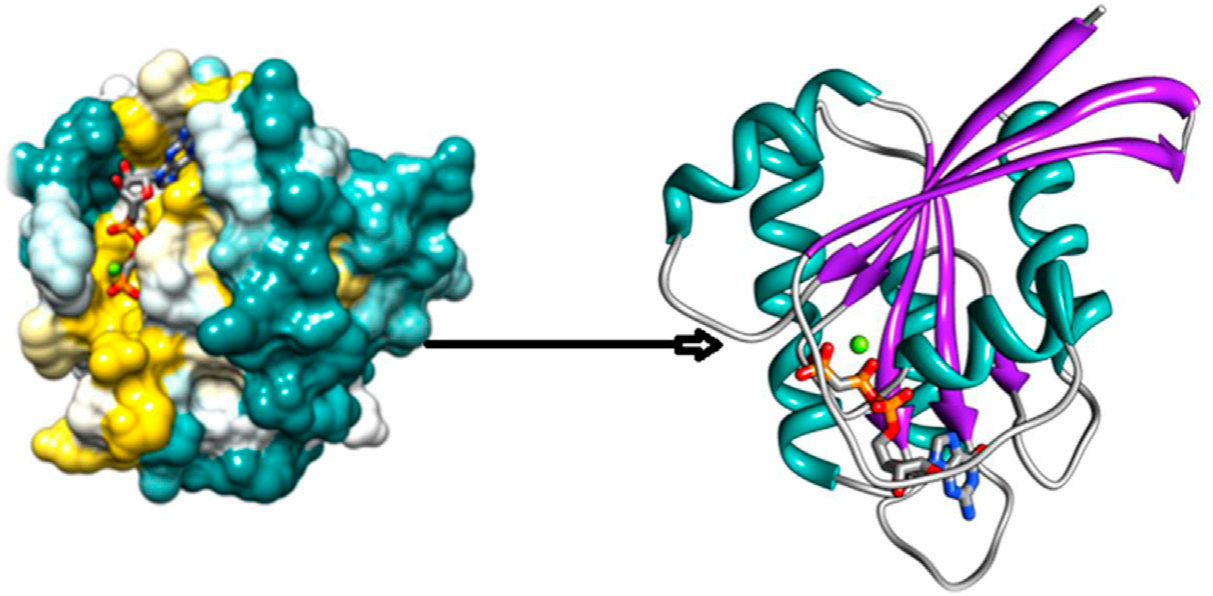
3D Structure of HRAS protein.

The Protein Data Bank (https://www.rcsb.org/) provides an extensive collection of experimentally determined protein structures that can be used for structural analysis (Protein Data Bank, 2019). In this study, we obtained the wild-type entire structure of the HRAS protein with its PDB ID: (6MQT) from the PDB. The protein structure was analyzed to identify features such as active sites, protein-protein interface sites, domain motifs, and ligand-binding affinities. The three-dimensional structure of HRAS was visualized in Figure 2, where the protein’s helices, beta-sheets, and coils were represented by yellow, cyan, and green colors, respectively. The structure of HRAS showed that it consists of five alpha-helices and six beta-strands, which are arranged in a characteristic fold called the G-domain as per earlier researcher investigations (Korzeniecki and Priefer, 2021). The G-domain contains the nucleotide-binding site, which is responsible for the hydrolysis of GTP to GDP, and plays a crucial role in regulating HRAS activity (Liu et al., 2019).

The protein structure was further analyzed to identify potential active sites and protein-protein interface sites, which can be targeted for drug design as per the results of researchers (Velazquez et al., 2018; Lin et al., 2020). In addition, the ligand-binding affinities of HRAS were predicted to identify potential small molecule inhibitors that can be used to target the protein in various diseases. The three-dimensional structure of HRAS provides essential insights into this protein’s function and regulation. It can be used to guide the design of new therapeutics for the treatment of HRAS-related diseases, as shown in an earlier exploration by Ahmad et al. (2022).

### 3.8 Mapping of most deleterious nsSNPs on HRAS gene

**FIGURE 3:**
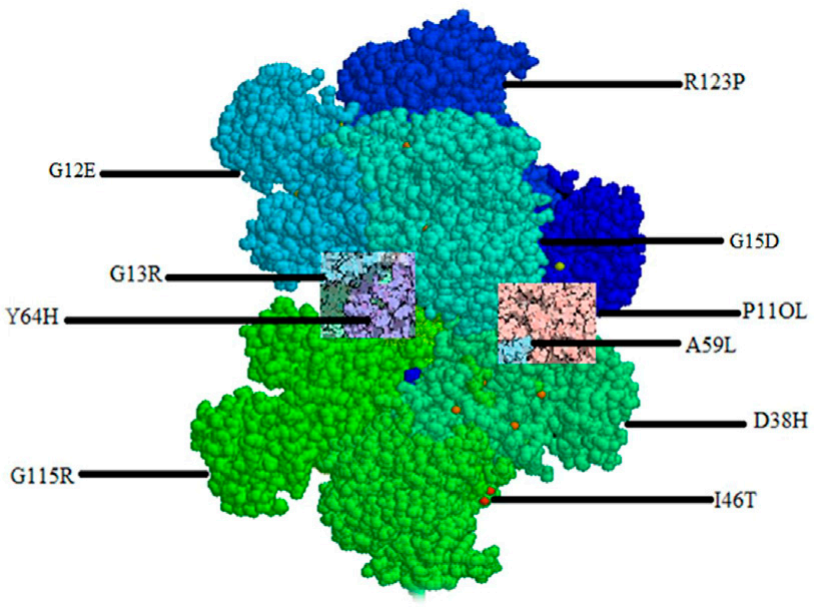
Mapping of most deleterious nsSNPs.

The mapping of the 10 most deleterious nsSNPs on the HRAS protein structure using mutagenesis techniques in Pymol software (as shown in Figure 3) provides a visual representation of the location and distribution of these mutations on the protein. The mutations were distributed throughout the protein structure, with several mutations located in the Group-I (G12E, G13R, G15D) and Group-II (Q61L, A59L) regions, which are known to play a crucial role in HRAS activation. Mutations in these regions can potentially lead to impaired GTP hydrolysis, affecting the normal functioning of HRAS. Moreover, several mutations were located in or near the protein’s active site (D38H, G60D, G60V), which could interfere with HRAS’s ability to interact with its downstream effectors and may impair its biological functions. The mutagenesis techniques used in Pymol also showed that some of the mutations (I46T, D38H, A59L, G60V) could form hydrogen bonds with neighbouring residues, suggesting a possible alteration of the protein’s conformation and potential effects on protein stability. Mapping these deleterious mutations in HRAS protein structure provides insights into how these mutations could potentially impact the protein’s structure and function, providing a foundation for further experimental investigation.

### 3.9 Structural analysis of HRAS protein

**FIGURE 4:**
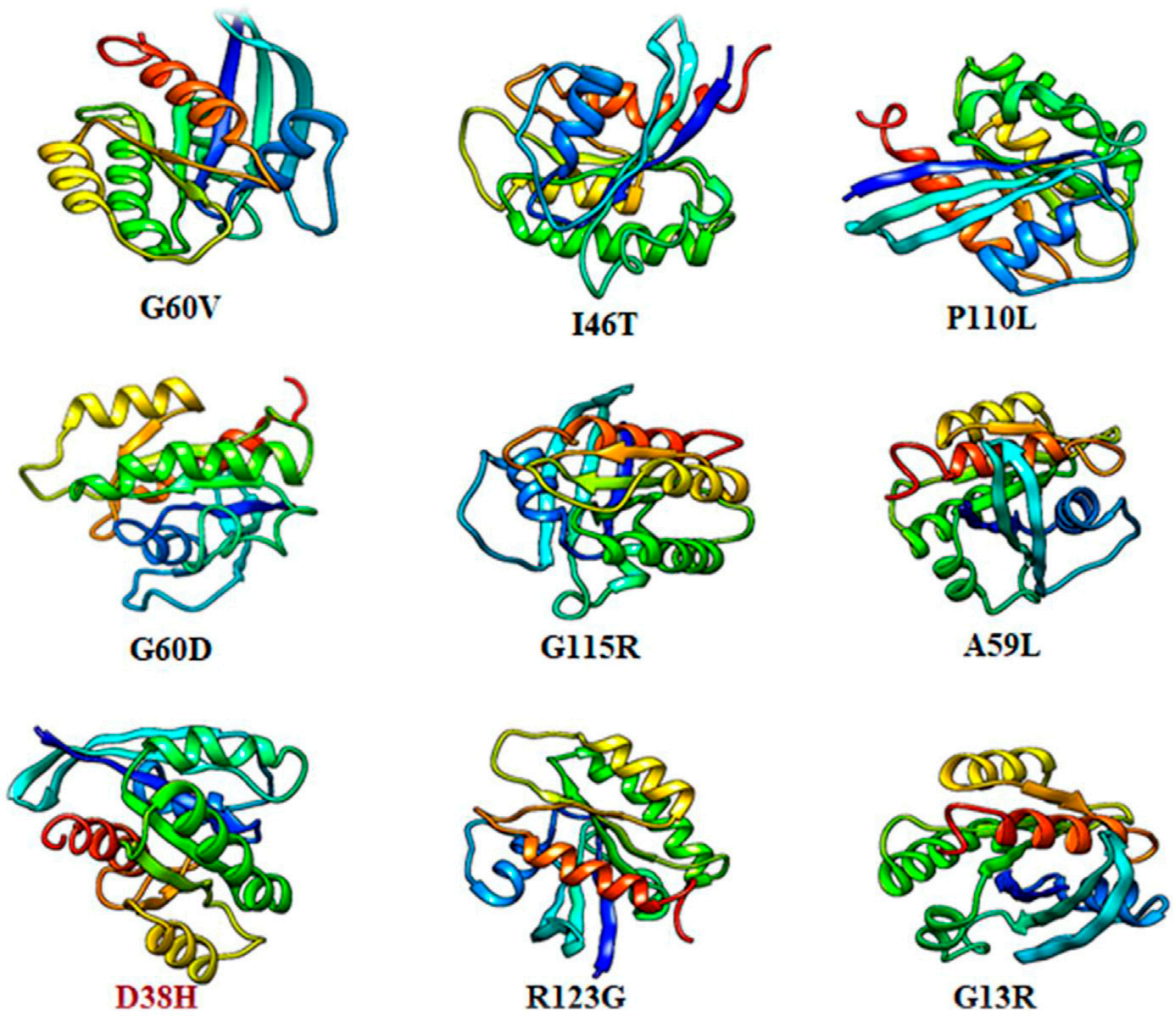
Muatantstructure of HRAS protein.

The native complete structure of HRAS was retrieved from the Protein Data Bank, and the Swiss Model server (https://swissmodel.expasy.org/) was used to predict the mutated form using homology modelling approaches. The ten most deleterious mutations, indicated by all the analyzing tools, were mapped in their respective region of HRAS using mutagenesis techniques in Pymol software, as shown in Figure 4. The mutated models were generated to further investigate the effect of these mutations on the HRAS protein structure, and energy minimization was carried out using Schrodinger. The energy minimization process minimized the energy and force acting on each atom in a gathering of atoms to obtain the most thermodynamically stable HRAS structure. The final and stable model of HRAS had an energy value of −18,756 kj/mol, which was significantly lower than the energy value of the initial model, which was −108915 kj/mol. This indicates that the mutated models had a more stable conformation than the initial models, after the energy minimisation process.

The deleterious mutated models of HRAS, including rs730880460 (G60V), rs730880460 (G60D), rs730880464 (R123P), rs750680771 (D38H), rs1564789700 (I46T), rs917210997 (G115R), rs369106578 (R123G), rs1204223913 (P11OL), rs727504747 (A59L), and rs104894228 (G13R), were generated using Pymol software and compared with the native HRAS protein structure. The mutations were observed to cause structural changes in different regions of HRAS, and the energy minimization process helped achieve more stable configurations. The results suggest that these mutations could potentially affect the function of HRAS and contribute to cancer development.

Each mutation can have different effects on the structure and function of the protein. The specific effects of each mutation can depend on a variety of factors, such as the location of the mutation in the protein, the surrounding amino acid residues, and the protein’s function as per the investigation of the earlier researcher (Cain et al., 2020). For example, the G60V mutation, predicted to be deleterious in our analysis, is located in the Group-II region of the protein and can cause problems in protein folding due to the larger size of the mutant residue compared to the wild-type residue. This can prevent the mutant residue from fitting correctly in the core region of the protein, potentially leading to the destabilization of the protein structure.

Similarly, the G115R mutation, also predicted to be deleterious, can lead to an incorrect conformation and disturbance of the local structure of the protein due to the larger size of the mutant residue, as a result, compare with (Wang S et al., 2022), and this result in loss of protein function. The G13R mutation located in the G-domain in the G-domain can cause loss of interaction because the mutant residue is more minor and has a different hydrophobicity compared to the wild-type residue as per earlier researchers (O’Bryan, 2019). This can affect the interaction between HRAS and its downstream effectors proteins, potentially leading to downstream signalling defects. The D38H mutation can cause loss of interaction or repulsion due to changes in charge, as the positively charged histidinepositively charged histidine replaces the negatively charged aspartic acid replaces the negatively charged aspartic acid. This can affect the interaction of HRAS with its upstream activators, potentially leading to downstream signaling defects.

### 3.10 Molecular docking of HRAS

Molecular docking is a computational method used to predict the binding mode and affinity of small molecules to a protein as per earlier researchers (Luo et al., 2019). In this study, molecular docking was applied to understand the effect of three deleterious mutations, G60V, G60D, and D38H, on the binding pocket of the HRAS protein. The HRAS protein structure with PDB ID: 6MQT was imported into MOE software (https://www.chemcomp.com/Products.htm), and the docked complexes of the wild-typeand mutated protein with ligands were generated. The docked complexes were analyzed for their docked score, hydrogen bonds, and pi-interactions within the 4.5 Å. The active binding site residues GLU62, GLY10, THR58, ASP33, ILE36, GLU63, TYR64, ALA11, TYR96, GLY13, and LYS16 were identified to be involved in pi-interactions and generate hydrogen bonds. The docked complexes showed significant binding affinity and interactions with the active binding site residues.

**FIGURE 5:**
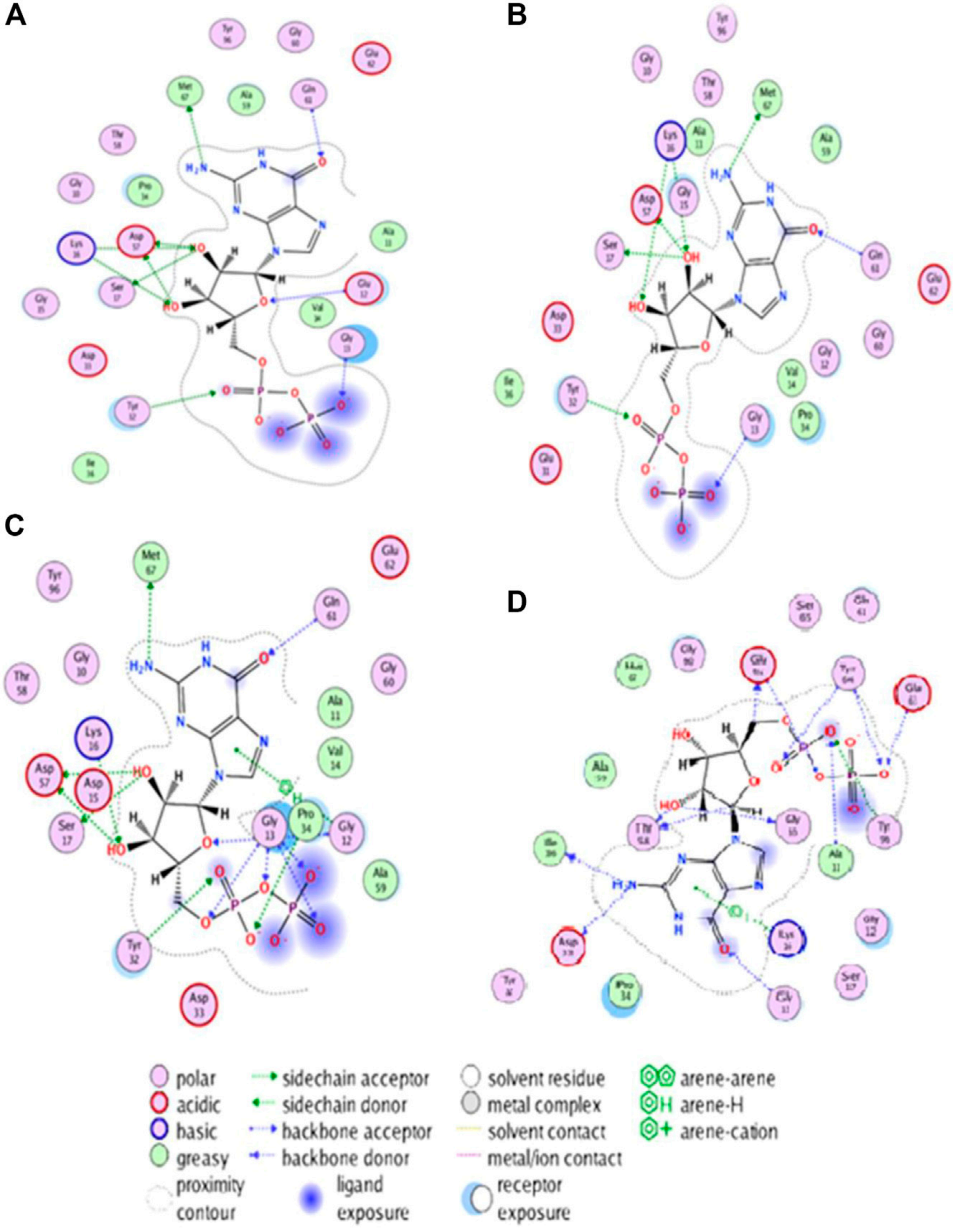
Visualizing structural changes in active site of HRAS protein due to mutations: Insights from hydrogen bonding and hydrophobic interactions.

When the three deleterious mutations, G60V, G60D, and D38H, were docked into the same binding pocket, the D38H residue was found to be involved in the binding interactions. With G60V, three hydrogen bonds were generated by the D38H residue, while one hydrogen bond with D38H and two with G60D were observed. This suggests that the D38H residue plays an important role in stabilizing the conformation of the mutated HRAS protein and results were compared with the investigation of the earlier researcher (Chai et al., 2022). The docked complexes were further subjected to MD simulation to analyze the stability and conformation of the wild-type and mutated complexes as shown in Figure 5. The MD simulation analysis revealed that the wild-type and mutated complexes were stable during the simulation. The RMSD and RMSF values were calculated, and it was observed that the mutated complexes had higher RMSD and RMSF values compared to the wild-type complex. This indicates that the mutated complexes had a slightly different conformation than the wild-type complex.

The -protein residues are shown as sticks, and the ligands are shown in space-filling mode. The wild-typel protein and the three mutated proteins are shown in different colors. The two-dimensional plot of interacting target residues shows the residues of the protein that areprotein residues involved in important interactions with the ligands. The residues involved in hydrogen bonding, Pi, and hydrophobic interactions are shown as circles in different colors. The Non-mutant protein is shown in blue color, and the residues involved in interactions with the ligands are labelled with their residue numbers. The plot in Figure 5 shows that the residues involved in interactions with the ligands are distributed throughout the protein’s active site. The residues involved in hydrogen bonding are mainly located in the loops and helices of the protein. The residues involved in Pi interactions are primarily situated in the helices and strands of the protein. The hydrophobic residues are mainly located in the core of the protein as mentioned in Figures 5A-D. For the G60V mutation, the plot shows that the mutation affects the hydrogen bonding with residue D38H and G60D mutation; the plot shows that the mutation affects the hydrogen bonding with residue D38H and generates two new hydrogen bonds with residue G60D. In D38H mutation, the plot shows that the mutation affects the hydrophobic interactions with the residues in the core of the protein. The plot provides valuable insights into the specific residues of the protein involved in the interactions with the ligands and how the mutations affect these interactions.

**FIGURE 6:**
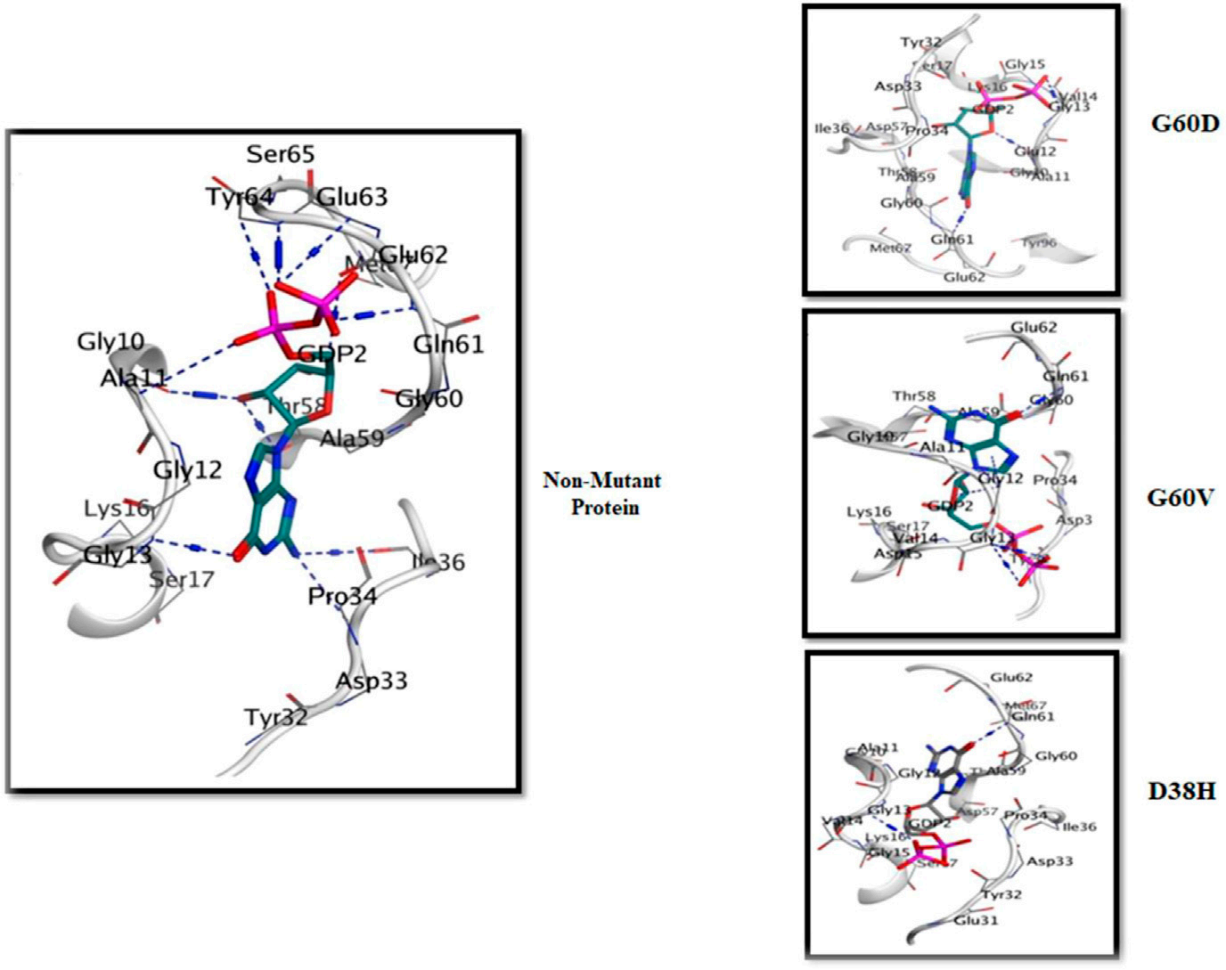
The 3D interaction of the docked complexes (G60V, G60D and D38H with Non-mutant protein).

The 3D interaction study of the docked complexes of G60V, G60D, and D38H with Non-mutant protein was performed to visualize the interactions and understand the binding mechanism of the mutated proteins with the Non-mutant protein as shown in Figure 6. The results showed that the mutated residues interacted with different residues compared to the Non-mutant protein. In the case of G60V, the mutated residue interacted with residues GLY13, ILE36, and GLU63. The GLU63 precipitate, which was involved in Pi-interactions and hydrogen bonding in the Non-mutant protein, did not form any interactions with the G60V mutated residue. Instead, GLY13 and ILE36 residues formed new interactions with the G60V mutated residue. This suggests that the G60V mutation might have altered the interaction pattern in the active site of the HRAS protein, which could affect the protein function.

In the case of G60D, the mutated residue interacted with residues ALA11, GLY10, and TYR96. The ALA11 residue, involved in Pi-interactions and hydrogen bonding in the Non-mutant protein, formed new interactions with the G60D mutated residue. Similarly, GLY10 and TYR96 residues also included new interactions with the G60D mutated residue. This indicates that the G60D mutation might have altered the binding pattern of the HRAS protein with ligands. In the case of D38H, the mutated residue interacted with residues GLU62, GLY10, and TYR64. The GLU62 residue, involved in Pi-interactions and hydrogen bonding in the Non-mutant protein, formed new interactions with the D38H mutated residue.

Similarly, GLY10 and TYR64 residues also formed new interactions with the D38H mutated residue. This suggests that the D38H mutation might have affected the ligands’ binding in the HRAS protein’s active site. Overall, the 3D interaction study revealed that the mutated residues interacted with different residues compared to the Non-mutant protein, which could affect the protein-ligand interactions and the function of the HRAS protein.

### 3.11 Molecular dynamic simulations

**FIGURE 7:**
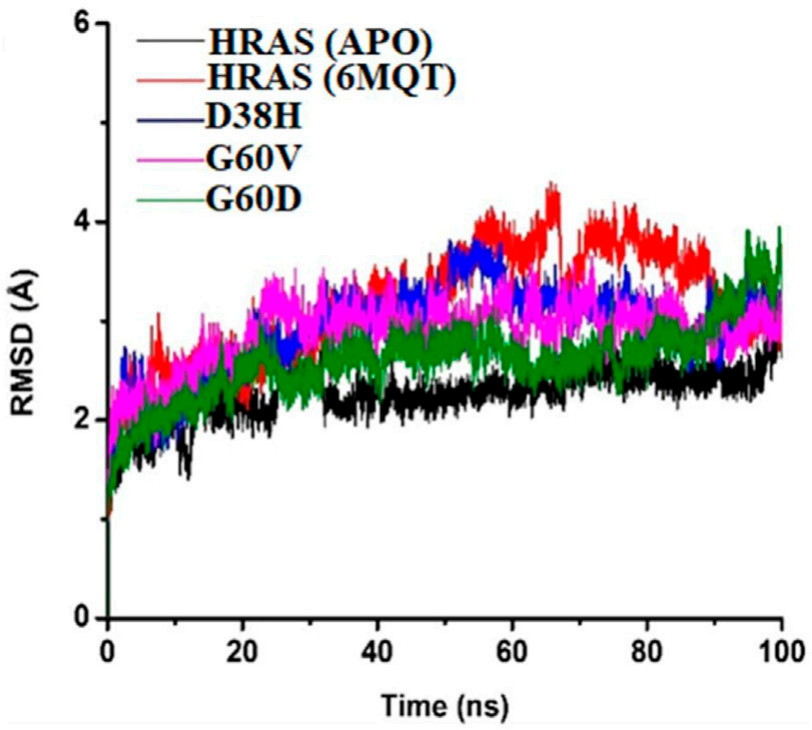
The RMSD of the APO protein was The Most Stable among All the Protein Models, with a Value of 3.14 Å as Shown in Figure 7.

The RMSD analysis is an essential tool for understanding the structural stability of a protein-ligand complex over a given time interval. The present study focused on analyzing the root-mean-square deviation (RMSD) values of protein-ligand complexes of HRAS in both APO and docked states, to examine the impact of three mutations, namely, G60V, G60D, and D38H, on the stability of the complexes. The findings of this study revealed that the wild-type HRAS complex demonstrated a greater degree of fluctuation compared to both the APO and mutated complexes. These results provide valuable insights into the effects of specific mutations on the stability of protein-ligand complexes and contribute to a better understanding of the dynamics of HRAS proteins in different states. The mean RMSD value of the wild-typecomplex was 4.40 Å, indicating that the protein underwent secondary structure changes with high loop regions compared to the average RMSD values of the mutated models. The mutated models (G60V, G60D, and D38H) decreased mean square calculation upon mutation, indicating a more stable environment and compactness of the whole system. The RMSD of the apoprotein was the most durable among all the protein models (Roy et al., 2022), with a value of 3.14 Å, as shown in Figure 7.

The results suggest that the mutations in the HRAS protein can lead to a more stable protein-ligand complex structure. The RMSD analysis also revealed that the ligand remained fixed throughout the simulation time interval, indicating that the mutations did not cause any significant changes in the ligand’s position or displacement. The RMSD analysis supports the conclusion that the mutations have a stabilizing effect on the protein-ligand complex. The minor fluctuations observed in the wild-type complex may indicate a higher degree of flexibility, potentially leading to changes in the protein-ligand complex’s structural framework.

**FIGURE 8:**
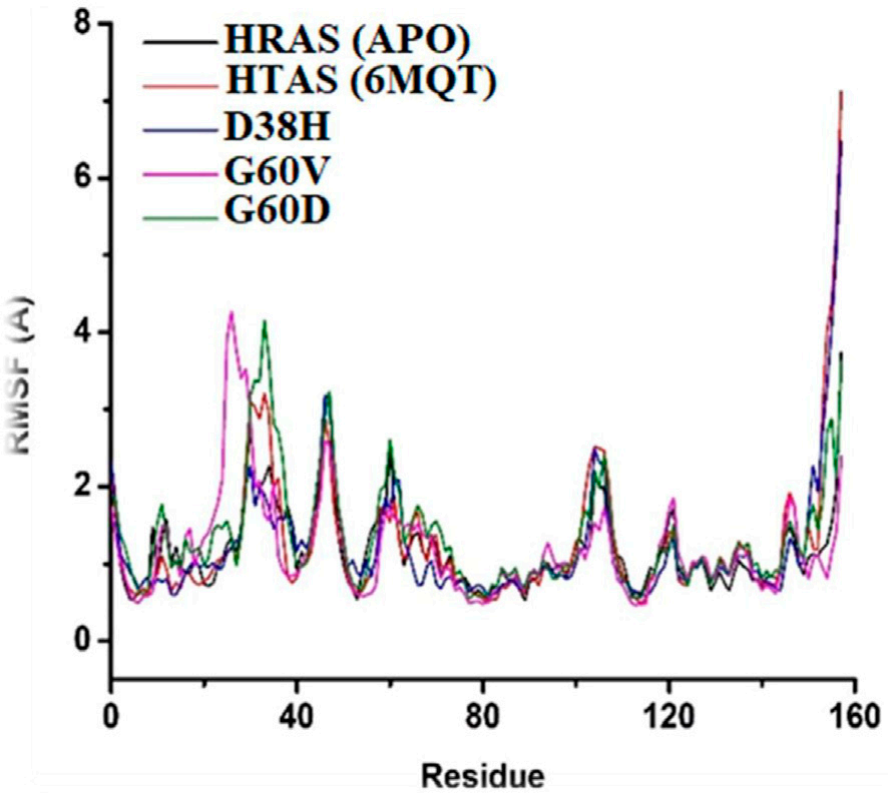
Conformational changes in structure that occurred in the docked protein complex at different time intervals.

The RMSF plot was generated to understand the fluctuation and stability of protein residues over the simulation period of 100 ns. The RMSF graph showed that the residues of the normal HRAS complex exhibited higher instability in comparison to the mutated and APO models, indicating more flexibility and conformational changes in the normal complex. The residues that showed higher RMSF values in the wild-type HRAS complex include Ser157, G12E, ILE46, and ASP47, which experienced significant structural changes concerning APO protein. On the other hand, the mutated complexes (G60V, G60D, and D38H) showed decreased RMSF values compared to the wild-type HRAS complex, indicating higher stability and less conformational changes, which are shown in Figure 8. The residues that exhibited high RMSF values in the mutated models were similar to those in the wild-type HRAS complex, including ILE46 and ASP47, but with lower fluctuations.

Notably, the residues ASN26 and ASP33 of the mutated complexes showed higher RMSF values than the wild-typw HRAS complex, indicating more fluctuations and conformational changes. These residues also showed significant structural changes during the simulation time, which may affect the stability and binding affinity of the mutated HRAS complex with ligands. The RMSF plot indicated that the mutated HRAS complexes were more stable than the wild-type complex, as evidenced by the lower RMSF values. However, some residues still experienced fluctuations and conformational changes, which could affect the stability and functionality of the protein-ligand complex.

The RMSF analysis provides information about the flexibility and fluctuation of each residue in the protein structure. The RMSF analysis revealed that the fluctuations occurred at the residual level, leading to the s system stability. The RMSF values showed changes occurring in regions other than the active site, which resulted in relatively more significant fluctuations. The loops in the protein structure are flexible regions, and upon ligand binding during MD simulations, they start changing their configuration at different intervals. The fluctuations in the loops and other flexible regions can be observed in the RMSF analysis. It can provide valuable information about the changes in the protein structure that occur during the simulation.

**FIGURE 9:**
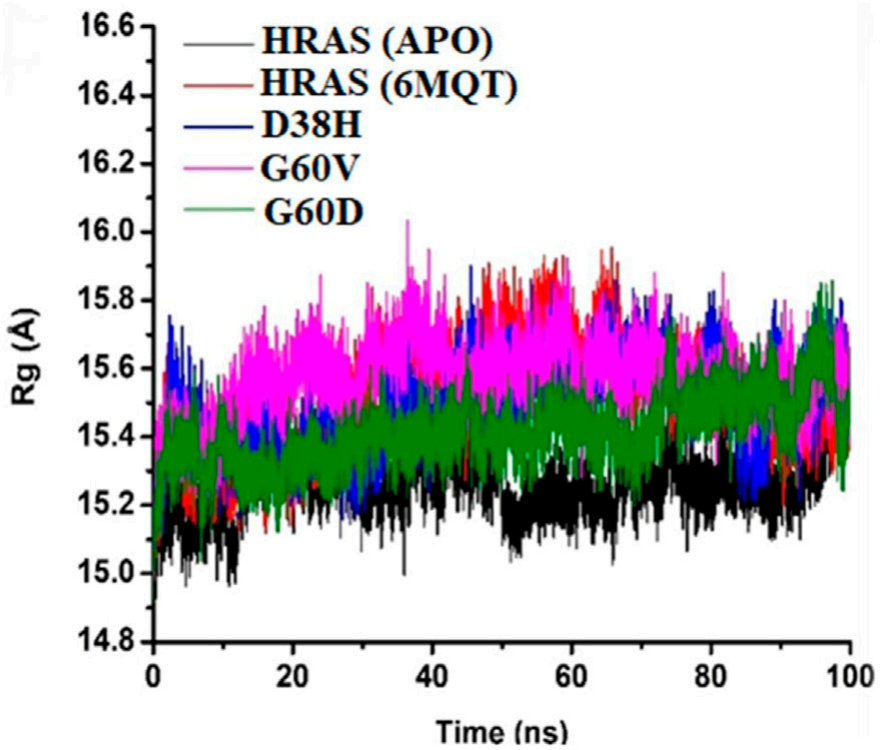
The protein Structure’s Stability throughout Simulation Time Interval.

The radius of gyration measures the compactness of the protein structure (Ahmed et al., 2020). It provides information about the average distance of all the atoms in the protein structure from the centre of mass. In this study, the radius of gyration analysis showed that the protein structure remained stable throughout the simulation time intervals. Although the radius of gyration values did not show any significant difference among all the proteins, it still provided helpful information about the compactness of the protein structure. The RMSF and radius of gyration analyses offered valuable information about the stability and structural changes in the protein-ligand complex during the MD simulations as mentioned in Figure 9. These analyses can be helpful in understanding the dynamic behaviour of the protein-ligand complex and provide insights into the binding mechanism of the ligand to the protein target.

The beta (B)-factor, also known as the temperature factor, measures a protein’s thermal stability and flexibility as per earlier researcher (Mao et al., 2020). It is calculated from the atomic displacement parameters obtained from X-ray crystallography experiments. The B-factor reflects a protein’s atomic vibrations and thermal motion, with higher values indicating greater mobility and lower stability (Wang W et al., 2022). The B-factor is often used to identify regions of a protein that are flexible or disordered (Vander Meersche et al., 2021). The root-mean-square fluctuation (RMSF) is another measure of a protein’s thermal stability and flexibility, based on the atomic changes of a protein over time (Khan et al., 2021). It indicates the amount of localized atomic fluctuations in a protein, which contribute to its overall vibration movement and thermal stability.

**FIGURE 10:**
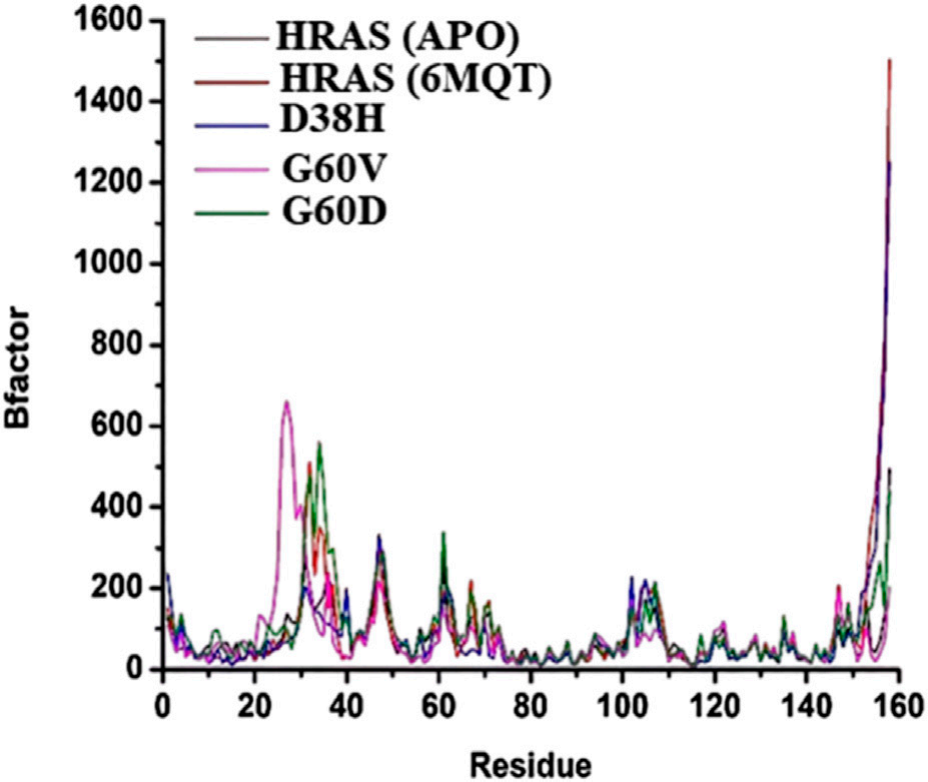
The beta (B)-factor, also known as the temperature factor, measures the thermal stability and flexibility of a protein.

The study used the B-factor and RMSF to investigate the thermal stability and flexibility of different HRAS models, including the APO (no ligand bound), wild-type, and mutant (G60V, G60D, and D38H) HRAS models. The average B-factor values were determined for each model, and the RMSF results were compared with the B-factor data. The results showed that the average B-factor values were highest for the G60D and wild-type HRAS models, indicating that these proteins had greater mobility and lower stability as mentioned in Figure 10. The lowest B-factor value was observed for the apo HRAS model, suggesting that this protein was the most stable. The G60V and D38H HRAS models had intermediate B-factor values, indicating moderate thermal instability.

**FIGURE 11:**
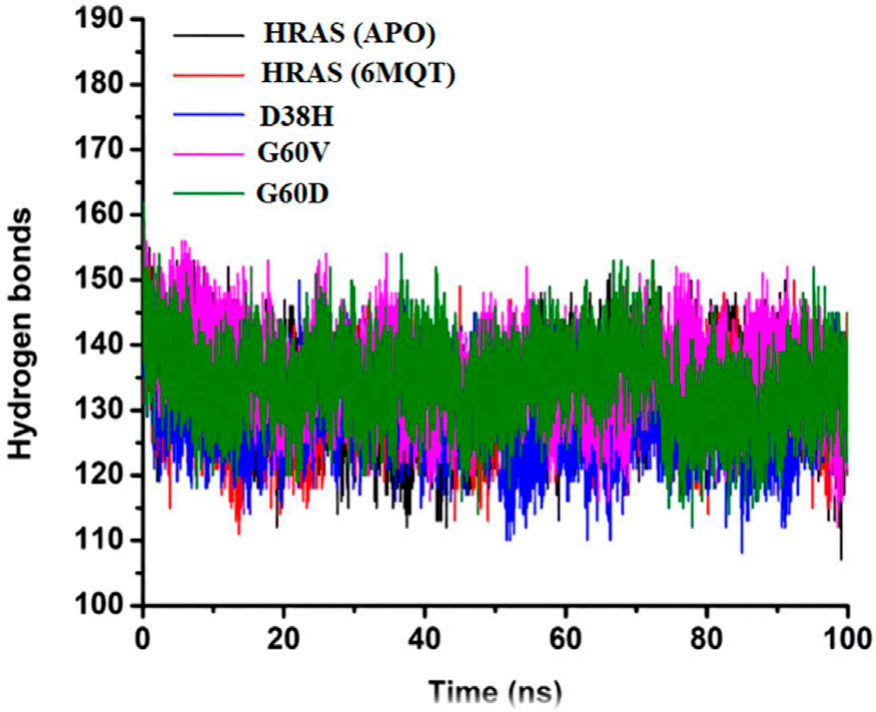
Presenting the number of hydrogen bonds of all three complexes with the time interval of 100 ns.

The RMSF results also showed that the thermal instability was higher at specific residues in the different HRAS models. These results were consistent with the B-factor data, as shown in Figure 11. In particular, residues 7, 13, 27, 38, and 50 had higher RMSF and B-factor values, indicating that these residues were more flexible and less stable in all the HRAS models. The study suggests that the B-factor and RMSF analyses can provide valuable insights into proteins’ thermal stability and flexibility. The results can be used to identify protein regions that are likely to be flexible or disordered, which could be important for understanding protein structure and function and drug design and development.

Hydrogen bonds are weak interactions between a hydrogen atom bonded to an electronegative atom (such as oxygen or nitrogen) and another electronegative atom in a nearby molecule (Karas et al., 2020). In biological systems, hydrogen bonds play a crucial role in determining the specificity and directionality of molecular recognition between molecules such as proteins, nucleic acids, and carbohydrates (Vladilo and Hassanali, 2018). In the study, the number of hydrogen bonds in the APO protein and all four HRAS complexes (including the G60V, G60D, and D38H mutants) were analyzed over time. The average number of hydrogen bonds was recorded for each system, and the results were presented in a time-dependent manner. The goal was to test the degree of intermolecular association across the simulation period and to investigate the effect of mutations on hydrogen bonding in the active site of the HRAS protein.

The results showed that the average number of hydrogen bonds was highest for the D38H mutant (138.9) and lowest for the G60V mutant (127.5), with the APO protein (133.6) and G60D mutant (136) having intermediate values. This suggests that the mutations can affect the number of hydrogen bonds formed within the active site of the HRAS protein. The hydrogen bond analysis also revealed that the number of hydrogen bonds decreased upon the D38H mutation and increased significantly upon the G60V, G60D, and D38H mutations. This suggests that the modifications can affect the hydrogen bonding patterns within the active site of the HRAS protein and potentially alter its structure and function. Figure 11 visually represents the number of hydrogen bonds formed in all three complexes over time. The data in the figure shows that the number of hydrogen bonds fluctuates over time, indicating that the interactions between the molecules are dynamic and can change with time. The analysis of hydrogen bonding patterns in the HRAS protein provides valuable insights into the molecular recognition and specificity of the protein. The results suggest that mutations can significantly affect the number of hydrogen bonds formed in the protein’s active site, which could impact its function and potentially lead to disease.

### 3.12 MM-GBSA analysis

In molecular docking and drug design studies, it is essential to estimate a ligand’s binding affinity to its target protein. One commonly used method for calculating binding energies is the molecular mechanics/generalized born surface area (MM-GBSA) approach (Pang et al., 2021). This method combines molecular mechanics force fields with implicit solvent models and can provide estimates of the relative binding free energies of different ligands to a target protein. In the study mentioned, the MM-GBSA approach was used to calculate the binding energies of the G60V, G60D, and D38H mutants and the normal HRAS protein. The results showed that all four systems had highly favourable binding energies, with the mutants having the highest. The table presented in the study (Table 3) showed that the binding energies for the G60V, G60D, and D38H mutants were −107.09, −109.42, and −107.18 kcal/mol, respectively. The Wild-type HRAS protein had a binding energy of −105.85 kcal/mol.

**TABLE 3:** Summary of the calculated binding free energies utilizing the MM/GBSA algorithm.

Protein complex	Coulomb solute	Coulomb solvent	VDW solute	VDW_Solvent	ΔG binding	Solvent GB
G60V	−4,908.6	−637.9	−299.437	−448.99198	−5,067.99	−633.999
G60D	−4,909.7	−685.6	−298.622	−86.0249567	−5,068.6	−683.333
D38H	−4,723.4	−620.7	−296.619	−80.1992073	−4,882.6	−620.903
HRAS-Normal	−4,758.06	−681.1	−144.27	−383.163312	−4,902.33	−678.231

In addition to examining the RMSD values, this study delved deeper into the binding energies of the protein-ligand complexes by scrutinizing their components. This comprehensive analysis facilitated a meticulous investigation of the specific interactions that play a role in determining the overall binding affinity, including van der Waals forces, electrostatic interactions, and hydrogen bonding. The outcomes of this investigation furnished valuable insights into the fundamental nature of the protein-ligand interactions, unravelling the intricacies of the underlying mechanisms that govern the binding affinity of these complexes. These findings have far-reaching implications for developing novel therapeutics targeting HRAS and allied proteins. An enhanced understanding of the binding energies can optimize drug efficacy and specificity and pave the way for developing more potent and targeted treatments. The results showed that the favourable Van der Waals and Coulombic interactions were the main contributors to the high binding energies in all four systems. Van der Waals interactions are attractive forces between atoms and molecules nearby, while Coulombic interactions are electrostatic forces between charged particles. The study also noted that Coulombic interactions reduced the binding affinity of some ligands towards the active site residues. This is because electrostatic repulsion between negatively charged ligands and negatively charged residues in the active site can decrease the strength of the binding. MM-GBSA calculations performed in this study provided insights into the binding energies and components of the G60V, G60D, and D38H mutants, as well as the normal HRAS protein.

Table 3 illustrates the prime MM-GBSA calculations for the G60V, G60D, and D38H mutants and the normal HRAS protein. The results exhibit various constituents of the binding energies, comprising Coulomb solute, Coulomb solvent, VDW (van der Waals) solute, VDW solvent, ΔG (change in Gibbs free energy) binding, and Solvent GB (Generalized Born) energy. Coulomb solute and Coulomb solvent indicate the electrostatic interaction energies between the protein and solvent molecule. In contrast, VDW solute and VDW solvent imply the van der Waals interaction energies between the protein and solvent molecules. ΔG binding represents the overall binding energy of the protein-ligand complex, which shows the free energy change upon binding the ligand to the protein. Solvent GB energy reflects the energy contribution from the solvent Generalized Born model.

The outcomes manifest that all four systems exhibit highly favourable binding energies, with the mutants displaying slightly higher binding energies than the normal HRAS protein. This suggests the mutations could intensify ligands’ binding to the protein’s active site. The Coulomb solute and Coulomb solvent energies were identified to be the highest in all four systems, indicating that electrostatic interactions play a significant role in the binding of ligands to the protein. The VDW solute and VDW solvent energies were also substantial, with the VDW solvent energy being the highest for the normal HRAS protein. This suggests that the solvent molecules have a part in stabilizing the protein-ligand complex. Regarding the particular mutants, the G60D mutant was observed to have the highest Coulomb solvent energy, while the D38H mutant had the lowest Coulomb solute and VDW solute energies. This implies that the mutations may affect the electrostatic and van der Waals interactions between the protein and solvent molecules, potentially influencing the binding of ligands to the active site.

The ΔG binding free energies of all the systems, including the wild-typesystem, were determined to be quite favourable, ranging from −4,902.33 to −5,068.6 kcal/mol, indicating that the binding of ligands to the HRAS protein is energetically stable. The favourable binding energies were due to the highly favourable Coulombic and Van der Waals interactions between the ligands and the protein. The Coulombic interactions between the ligands and the protein were more favourable in all the systems, resulting in lower binding energies. The electrostatic solvation energy of Generalized Born was compensated by the Coulombic interactions, which were less favourable. This suggests that the Coulombic interactions play a significant role in ligand binding towards the active site residues in the HRAS protein.

All four complexes displayed a clear pattern, which was considerably similar, indicating that the mutations did not significantly affect the binding energy. This observation is intriguing and prompts further investigation into the conformational characteristics of the systems, i.e., the interaction between the ligands and the receptor. The molecular docking and dynamics results may explain the *in vitro* findings logically. Overall, the MM-GBSA calculations provide valuable insights into the thermodynamic stability of the ligand-protein complex, which is crucial for understanding the binding affinity and specificity of the ligand towards the protein. The favourable binding energies and strong Coulombic interactions observed in this study suggest that the ligands are likely to bind tightly to the active site residues of the HRAS protein, which may have implications for the design of novel HRAS inhibitors with improved efficacy and specificity.

### 3.13 3D modeling and structural analysis of HRAS protein

The study aimed to investigate the impact of mutations on the structure and stability of the HRAS protein. To achieve this, the native structure of the HRAS protein was obtained from the Protein Data Bank (PDB), and homology modeling approaches were used to predict the mutated structure of the protein. The mutations were identified using various Single Nucleotide Polymorphism (SNP) databases, including rs730880460 (G60V), rs730880460 (G60D), rs730880464 (R123P), rs750680771 (D38H), rs1564789700 (I46T), rs917210997 (G117R), rs369106578 (R123G), rs1204223913 (P11OL), rs727504747 (A59L), rs104894228 (G13R), and rs1564789552 (Y64H). The PyMOL software was utilized to generate the mutated models of HRAS.

The energy minimization process was carried out using Schrödinger, a method that minimizes the energy and force load applied upon every atom in a gathering of atoms to obtain the best thermodynamically stable HRAS structure. The final and stable model of the HRAS protein was obtained after energy minimization, and the energy value was reported to be −17,755 kJ/mol, significantly lower than the initial energy value of −107916 kJ/mol. This indicates that the energy minimization process significantly improved the stability of the protein structure. The results of this study suggest that mutations can dramatically alter the structure and stability of the HRAS protein. The homology modelling approach successfully predicted the mutated models of the protein, and the energy minimization process further improved the stability of the predicted structures. The energy minimization process revealed a remarkable reduction in the energy value of the predicted protein structures. This significant decrease in energy strongly suggests that the systems are thermodynamically stable and likely to represent the native conformation of the protein. The observed reduction in energy value serves as a reliable indicator of the stability and accuracy of the predicted structures, bolstering confidence in the findings of this study.

The study is significant in providing insights into the impact of mutations on the stability of the HRAS protein. The mutated models generated in this study can be used for further studies to understand the effect of mutations on protein function and interactions. Overall, the results of this study provide a foundation for developing new HRAS inhibitors that can target the mutated forms of the protein with improved efficacy and specificity.

#### 3.13.1 Residue substitutions and HRAS protein function

In the case of R123P, substituting R with P at position 123 may result in a significant loss of interaction due to the marked disparity in size and hydrophobicity between the wild type and mutant residues. The smaller size of the mutant residue may significantly impede the protein’s interactions with other molecules. In D38H, the mutation of D to H at position 38 can potentially disturb the protein’s interaction with other parts of the protein. The loss of the charge of the wild type residue due to this mutation may significantly impact the protein’s functionality. In I46T, substituting I with T at position 46 can cause the core region of the protein to lose its hydrophobic interactions. This is because the mutant residue is, more minor, but more hydrophobic than thewild-type residue. In G117R, the mutation of G to R at position 117 could result in a protein folding problem. Introducing a charge in a buried region of the protein due to the mutant residue, which is normally flexible, may disrupt the local structure of the protein. In R123G, the mutation of R to G at position 123 could cause the protein to lose its ability to interact with other molecules due to the charge difference among mutant and wild-type res and. In G60V, substituting P with L at position 110 can result in the loss of external interactions with other proteins in a pathway due to the difference in size between the mutant and wild-type residues. The native residue is embedded in the core region, which may make it difficult for the more considerable mutant residue to fit. In A59L, the mutation of A to L at position 59 can potentially cause the mutant residue, which is larger than the wild-type residue, to not fit in the protein’s core region. This could result in a loss of external interactions. In G13R, substituting G with R at position 13 may significantly impede the protein’s interaction with other molecules. Introducing a charge due to the mutant residue may cause a repelling effect between the mutant and adjacent residues, which are typically found on the protein’s surface. In Y64H, converting Y to H at position 64 may result in a loss of external interactions at this point has potential result in the loss of hydrophobic interactions with other molecules.

## 4 Discussion

Single nucleotide polymorphisms (SNPs) are a type of genetic variation in the human genome that can result in amino acid substitutions, leading to alterations in protein structure and function (Stalin et al., 2022). Non-synonymous SNPs (nsSNPs) result in an amino acid substitution, and they have been implicated in many genetic disorders. Therefore, it is essential to understand the functional impact of nsSNPs on protein structure and function. Bioinformatics approaches have become an essential tool for predicting the practical impact of nsSNPs (Yazar and Özbek, 2021). These approaches can be broadly classified into two categories: *in silico* and experimental. Experimental approaches involve biochemical and biophysical techniques, such as X-ray crystallography, NMR spectroscopy, and site-directed mutagenesis, to directly measure the functional impact of nsSNPs. However, these methods are time-consuming, expensive, and require specialized expertise. In contrast, *in silico* approaches use computational methods to predict the functional impact of nsSNPs, making them a faster and more cost-effective way to screen for potentially deleterious variants.

SIFT (Sorting Intolerant from Tolerant) and PolyPhen-2 (Polymorphism Phenotyping v2) are widely used *in silico* tools for predicting the functional effects of nsSNPs. SIFT uses a sequence homology-based approach to predict the impact of an amino acid substitution on protein function. It compares the protein sequence of interest to a database of related protein sequences and determines the degree of conservation of the substituted amino acid. If the amino acid is highly conserved, it is predicted to be intolerant to substitution, and the variant is classified as damaging. PolyPhen-2, conversely, uses a combination of sequence- and structure-based approaches to predict the impact of amino acid substitutions on protein structure and function. It considers factors such as the physicochemical properties of the substituted amino acid, the local protein structure, and the conservation of the substituted amino acid across different species. Variants are classified as damaging if they are predicted to affect protein function, stability, or interaction with other molecules. Both SIFT and PolyPhen-2 have been shown to have high accuracy in predicting the functional impact of nsSNPs. However, they use different algorithms and approaches, which can lead to differences in their predictions. Therefore, it is recommended to use both tools to increase the accuracy of predictions. In addition, other tools, such as PROVEAN, MutPred, and MutPred-LOF, are also available and can be used in combination to improve the accuracy of predictions.

Other tools, such as SNP&GO, P-Mut, Phd SNP, and ROVEAN, have also been developed to predict the functional effects of nsSNPs. These tools use different algorithms and approaches to predict the impact of nsSNPs on protein structure and function. They can provide complementary information to other tools. In recent research, a comprehensive analysis of nsSNPs in the HRAS gene was performed using various *in silico* tools to identify potentially deleterious SNPs that may be associated with the disease. After searching the SNP databases, 50 hits were found, and the mutants’ rsIDs were submitted to SIFT and PolyPhen-2 for functional analysis. Of the 50 SNPs, 24 were found to be non-tolerable by SIFT, and 28 were predicted to be possible or probably damaging by PolyPhen-2. The results were then validated using other tools, and 11 SNPs were identified as deleterious by all of the tools. The impact of these deleterious SNPs on protein structure was analyzed using software such as PyMOL and SNP effect. In conclusion, *in silico* approaches and tools have become powerful tools for identifying and characterizing the functional effects of nsSNPs and predicting their potential association with various diseases. While no single tool can accurately predict the practical effects of nsSNPs, a combination of tools can provide more accurate and reliable predictions. Moreover, detailed analysis of the impact of deleterious SNPs on protein structure and function can provide important insights into the development of genetic disorders and may lead to the development of novel therapeutic approaches.

Bioinformatics is an essential tool for analysing genetic variations, including non-synonymous single nucleotide polymorphisms (nsSNPs) (Wang et al., 2020). In-silico approaches have been developed to predict the functional impact of nsSNPs on protein structure and function and to distinguish between neutral and deleterious variants. One of the widely used approaches for predicting the deleterious effect of nsSNPs is SIFT which predicts whether an amino acid substitution is likely to affect protein function based on sequence conservation. Another approach is PolyPhen (Polymorphism Phenotyping), which is a tool for predicting the functional effect of amino acid substitutions on protein structure and function (Seifi and Walter, 2018). It uses both sequence-based and structure-based features to predict the impact of a variant on protein function. In addition, machine learning-based methods such as Random Forest and Support Vector Machines (SVM) have also been developed to classify nsSNPs as deleterious or neutral (Ge et al., 2021). These methods use a combination of sequence, structural and evolutionary features to predict the functional impact of nsSNPs.

In a recent study by Behairy et al. (2022), various SNPs of the HRAS gene were assessed to identify those potentially deleterious and associated with disease development using computational approaches. We searched for nsSNPs against HRAS in SNP databases and found 138 hits. The rsIDs of these mutants were submitted to two widely used computational tools, SIFT and PolyPhen2, to determine the functional effects of the nsSNPs. SIFT identified 15nsSNPs as non-tolerable, while PolyPhen2 showed 23 nsSNPs as possible and probably damaging. To validate the results, the authors submitted the rsIDs of the 23 nsSNPs to several other tools, including SNP&GO, P-Mut, Phd SNP, and ROVEAN. Among these, 10 SNPs, with rsIDs of rs730880460, rs730880460, rs730880464, rs750680771, rs1564789700, rs917210997, rs369106578, rs1204223913, rs727504747, rs104894228, and rs1564789552, were identified as deleterious by all the tools. The authors highlighted that the association of these damaging nsSNPs with disease development has not been reported in any other study yet. Therefore, further research is required to validate the functional significance of these nsSNPs in HRAS and their association with disease development.

The authors emphasized that combining multiple algorithms frequently is a powerful tool for selecting candidate functional nsSNPs. In a previous study by Falahi et al. (2021), it was reported that among various Insilico tools, Polyphen 2 and SNAP show better performance for identifying functional nsSNPs. Thus, using multiple computational tools and integrating their results can provide more reliable predictions of functional nsSNPs, which can aid in understanding the molecular basis of diseases caused by nsSNPs. Overall, the study by Behairy et al. (2022), highlights the importance of using computational approaches to identify potential deleterious nsSNPs in genes and their association with disease development. The study also underscores the significance of using multiple computational tools to validate the functional relevance of nsSNPs and the need for further research to establish their association with disease development.

In our study, we utilized SNP effect to assess the impact of SNPs on the aggregation tendency, amyloid propensity, and chaperone binding of HRAS protein. The outcomes of SNP effect revealed that rs1204223913 increases the aggregation propensity of HRAS protein with a dTANGO score of 547.61, while rs1564789700 decreases the aggregation propensity with a dWALTZ score of −106.36. However, we observed that most of the variations did not affect the molecular phenotype of the protein. Although these variations may convey some damaging mutation on HRAS protein, they seem unrelated to the protein’s aggregation tendency, amyloid propensity, or chaperone binding tendency, according to the outcomes of the SNP effect. To further investigate the impact of deleterious nsSNPs on the protein structure of HRAS, we retrieved the tertiary structure of HRAS from PDB with PDB id (6MQT). We mapped all 10 deleterious nsSNPs using PyMOL software. Our findings indicate that these nsSNPs are located in different regions of the protein structure and may affect the protein function by altering its stability or interactions with other molecules.

Several previous studies have also investigated the impact of nsSNPs on protein structure and function. For instance, a survey by Taghvaei et al. (2021) utilized *in silico* tools to predict the effects of nsSNPs on protein function and reported that these tools aid in identifying deleterious nsSNPs associated with human diseases. Additionally, a study by Teng et al. (2009) utilized molecular dynamics simulations to investigate the impact of nsSNPs on protein stability and interactions. Our study highlights the importance of using computational approaches to identify deleterious nsSNPs and their potential effects on protein structure and function. Our findings also suggest that combining multiple *in silico* tools can provide a more accurate prediction of the functional impact of nsSNPs on proteins.

Using computational methods, our investigation aimed to detect harmful single nucleotide polymorphisms (SNPs) in the HRAS gene. By examining several SNP databases and employing different analytical tools, we identified 10 damaging SNPs. Our study is significant because mutations in HRAS are associated with various diseases, including cancer. Recognizing potentially harmful SNPs in HRAS can help us understand the genetic basis of these diseases and facilitate the development of personalized treatments. Moreover, using computational methods to identify functional nsSNPs can considerably reduce the time and cost required for experimental validation. Our findings emphasize combining various algorithms and tools to pinpoint candidate functional nsSNPs. In conclusion, our research offers a valuable contribution to the genetics field and can assist in developing personalized medical interventions.

## 5 Conclusion

We investigate the potential impact of nsSNPs in the HRAS gene on the structure and function of the HRAS protein, as well as their potential contribution to the development of various types of cancers. Specifically to identify and characterize nsSNPs within the coding region of HRAS that can cause detrimental mutations, disrupt normal protein function, and activate oncogenic signaling pathways. To decrease expenses and enhance the efficiency of genetic association studies, We applied *in silico* approaches, including SIFT analysis, PolyPhen2 scores, and TI scores, to predict the potential impact of rare genetic variants on HRAS protein function, and identified 50 nsSNPs in total, of which 23 were located in the exon region of the HRAS gene and were likely to be deleterious. Among these 23 nsSNPs, 10 had the most destructive impacts, including G60V, G60D, R123P, D38H, I46T, G115R, R123G, P11OL, A59L, and G13R, with DDG values ranging from −3.21 to 0.87 kcal/mol. We conducted molecular dynamics (MD) simulations to analyze the stability, flexibility, and compaction of the HRAS protein to investigate the consequences of specific non-synonymous single nucleotide polymorphisms (nsSNPs). Our predicted results indicated that the stable model of HRAS had a significantly lower energy value than the initial model, suggesting that these nsSNPs may alter the stability of the protein. Furthermore, we analyzed the binding energies of both the wild-type and mutant HRAS protein with docked complexes to understand the potential impact of these mutations on the activation of oncogenic signalling pathways. Our findings indicated that the G60V, G60D, and D38H mutants had higher binding energies than the wild-type HRAS protein, potentially leading to the activation of oncogenic signalling pathways and contributing to the development of various types of cancers. Our systematic study analysis provides essential insights into the potential functional role of nsSNPs in the HRAS gene in cancer development. It could inform future studies aimed at developing targeted therapies for cancer treatment.

## Data Availability

The datasets presented in this study can be found in online repositories. The names of the repository/repositories and accession number(s) can be found in the article/Supplementary Material.
